# Costs of a clinical pathway with point‐of‐care testing during influenza epidemic in a Dutch hospital

**DOI:** 10.1111/irv.12808

**Published:** 2020-10-12

**Authors:** Sierk D. Marbus, Suzanne P. M. Lutgens, Arianne B. van Gageldonk‐Lafeber, Eric H. L. C. M. Hazenberg, Mirjam H. A. Hermans, Anita W. M. Suijkerbuijk

**Affiliations:** ^1^ National Institute for Public Health and the Environment Bilthoven The Netherlands; ^2^ Jeroen Bosch Hospital ‘s‐Hertogenbosch The Netherlands

**Keywords:** clinical pathway, hospitalisation costs, influenza, point‐of‐care testing

## Abstract

Our study aim was to determine how a new clinical pathway, including PCR‐based influenza point‐of‐care test (POCT), influences the hospitalisation costs of patients suspected of influenza presenting at the emergency department of a Dutch hospital during two consecutive influenza epidemics (2016‐2017 and 2017‐2018). Compared to mean costs per patient of €3661 in 2016‐2017, the implementation of this new clinical pathway with influenza POCT in 2017 was associated with mean costs per influenza‐positive patient of €2495 in 2017‐2018 (*P* = .3). Our study suggests favourable economic results regarding a new clinical pathway with influenza POCT, reflecting a more efficient care of patients suspected of influenza presenting at the emergency department.

## 1 INTRODUCTION

Seasonal influenza epidemics cause substantial morbidity and lead to excess hospitalisations and mortality, especially in the elderly.^1^ A sudden increase in the number of patients requiring hospital care for severe acute respiratory infections (SARI), especially pneumonia as complication of influenza virus infection, may pose a significant burden for hospitals in managing bed and staff capacity.^2^ Whether a SARI patient is tested for influenza virus infection is the decision of the individual attending physician in most hospitals and mainly relies on laboratory‐based PCR testing with a turnaround time of 24‐48 hours.

More frequent testing and timely diagnosis of influenza may better guide isolations and improve patient flow through the hospital and thereby contribute to a more efficient management of patients.^3^, ^4^ In influenza season 2017‐2018, the Jeroen Bosch Hospital (JBH) in ‘s‐Hertogenbosch, the Netherlands, implemented a PCR‐based point‐of‐care test (POCT) for influenza virus type A and type B and respiratory syncytial virus (RSV) for all patients presenting with SARI at the Emergency Department (ED).^5^ Furthermore, a temporary ward dedicated specifically to care of influenza‐positive patients was established. The POCT (Cobas Liat Assay) has high sensitivity and specificity for influenza virus types A and B, and RSV, and can be performed by non‐laboratory personnel at the ED.^3^ The aim of this study was to determine how a new clinical pathway, including POCT, influences the hospitalisation costs of patients suspected of influenza presenting at the ED.

## 2 METHODS

### 2.1 Study population and period

The study population consisted of patients with an acute respiratory tract infection (RTI) presenting at the ED of a Dutch hospital (JBH) during two consecutive influenza epidemics (2016‐2017 and 2017‐2018). JBH is a large general hospital in the south‐eastern part of the Netherlands with 575 beds and catchment population for RTI of 323 000 persons.

### 2.2 Clinical pathways

In influenza season 2016‐2017, influenza diagnostics were requested at the discretion of the treating physician depending on the differential diagnosis, taking into account epidemiology, patient symptoms and costs. Patients suspected of an influenza virus infection by their treating physician had nose‐throat swabs collected and subsequently analysed with a laboratory‐developed real‐time polymerase chain reaction (PCR) test (LDT) for influenza virus types A and B on the BD MAX System. All patients with pending influenza test results were put in droplet isolation on the general ward or intensive care unit (ICU) until test results became available within 24 hours, after which a decision was made on whether isolation had to be continued or not.

In influenza season 2017‐2018, from 8 January 2018, a new clinical pathway for patients suspected of an influenza virus infection was implemented in JBH, consisting of three interventions: (a) clinical rule for influenza diagnostics in ED patients; (b) influenza POCT test (Cobas Liat, Roche Molecular Diagnostics); and (c) temporary influenza ward for cohort isolation. The clinical rule for requesting influenza POCT in ED patients was defined as having: (a) a temperature ≥38°C; and (b) symptoms of an acute RTI. As POCT, the influenza A, B and RSV real‐time PCR assay on the Cobias Liat System with a turnaround time of 20 minutes was used. The temporary influenza ward consisted of maximum 15 beds for influenza‐positive patients, excluding patients admitted to the ICU, haematology, oncology or paediatric ward. After isolation of maximum 5 days, the influenza‐positive patients were allocated to another ward or discharged home.

### 2.3 Clinical data for cost estimates

Retrospective data were collected using the electronic patient records. The dataset included number of requested influenza tests (LDT/POCT), admissions to hospital (ward/ ICU), isolated patients, treatment for patients suspected of influenza (antibiotics/antivirals), and median length of hospital stay (LOS). Costs were calculated from the start of the epidemic using a bottom‐up approach following Dutch guidelines for economic evaluations.^6^ Test costs were retrieved from JBH, ED consultations and hospitalisation costs were based on the national cost manual for economic evaluations,^7^ isolation costs were taken from literature,^8^ and medication costs were taken from the National Health Care Institute website.^9^ Hospitalisation costs were calculated by multiplying recorded units of used healthcare resource with corresponding unit prices (Table S1). The maximum isolation duration, additional diagnostics and type and duration of antibiotic and/or antiviral treatment were based on recent literature (footnotes Table 1). All costs were expressed in 2018 euros.

**TABLE 1:** Hospitalisation costs of influenza‐positive and influenza‐negative patients in a Dutch hospital during influenza epidemic 2016‐2017 and 2017‐2018

Cost type	Influenza test result	Influenza epidemic 2016‐2017 (week 48, 2016‐week 10, 2017)			Influenza epidemic 2017‐2018 (week 2, 2018‐week 15, 2018)		
		No. patients	Hospitalisation costs (€)		No. patients	Hospitalisation costs (€)	
			Per week	Per person		Per week	Per person
Emergency department^a^	positivenegative	189 402	3398 7227	270 270	624 922	9348 13 812	270 270
Admissions							
General ward^b^	positive negative	161 346	31 170 74 417	2904 3226	434 719	55 084 108 280	2285 2711
Intensive care unit^c^	positive negative	11 27	7692 48 712	10 489 27 062	21 20	12 237 15 384	10 489 13 846
Diagnostics							
Influenza test^d^	positive negative	189 402	1417 3013	112 112	624 922	3898 5759	112 112
Urine antigen test	positive negative	172 373	165 358	14 14	455 739	332 538	13 13
Other^e^	positive negative	189 402	1176 2502	93 93	624 922	3236 4781	93 93
Isolation^f^	positive negative	172 373	890 386	78 16	455 0	1963 0	78 0
Treatment							
Antibiotics^g^	positive negative	99 229	195 450	29 29	206 364	330 584	29 29
Oseltamivir^h^	positive negative	15 34	22 10	22 4	50 13	61 2	22 2
Total costs	positive negative	189 402	46 125 137 075	3661 5115	624 922	86 488 149 141	2495 2912

^a^ Costs for emergency department consultation.

^b^ Weighted mean hospitalisation costs per day on general ward.

^c^ Hospitalisation costs per day on intensive care unit including costs for diagnostics and medication.

^d^ Costs for influenza LDT (BD MAX System) in 2016‐2017 and influenza POCT (Cobas Liat) in 2017‐2018.

^e^ Other diagnostics include chest X‐ray, blood examination (Hb, MCV, Ht, leucocytes, platelets, ASAT, ALAT, GGT AF, LD bilirubin, creatinine, urea, sodium, potassium, chloride, calcium, total protein, glucose, CRP) urine examination (urine screening test).

^f^ Isolation costs in the general wardroom and associated isolation costs (use of gloves, Free Flight Phase 1 masks, additional workload for medical and cleaning personnel) for one day in influenza‐negative patients (only in 2016‐2017) and the duration of five days in influenza‐positive patients (2016‐2017 and 2017‐2018).

^g^ Costs for antibiotic treatment on the general ward were calculated for average empiric antibiotic therapy course in line with Dutch Working Party on Antibiotic Policy (SWAB) guidelines in Jeroen Bosch Hospital.

^h^ Costs for oseltamivir treatment on the general ward were calculated for the duration of five days according to SWAB guidelines.

## 3 RESULTS

The influenza epidemic of 2016‐2017 lasted 15 weeks (week 48 of 2016 until week 10 of 2017), while the duration of the influenza epidemic of 2017‐2018 was 18 weeks (week 50 of 2017 until week 15 of 2018). Compared to mean costs per patient of €3661 in 2016‐2017, the implementation of this new clinical pathway with influenza POCT in 2017 was associated with mean costs per influenza‐positive patient of €2495 in 2017‐2018 (Mann‐Whitney *U* test; *P* = .3). The mean costs per influenza‐negative patient were €5115 in 2016‐2017, and after the implementation of the new clinical pathway, this amounted to €2912 in 2017‐2018 (Mann‐Whitney U test; *P* = .8) (Table 1).

Sensitivity analysis indicated that ICU admissions affected mean cost estimates of influenza‐negative patients in 2016‐2017 compared to 2017‐2018 to a larger degree than influenza‐positive patients (Table S2). A higher number of ICU admissions (27 versus 20) and notably longer LOS in ICU (12.9 versus 6.6) of influenza‐negative patients contributed to this finding.

## 4 DISCUSSION

A new clinical pathway in JBH, including a clinical rule for requesting influenza diagnostics, POCT testing and establishment of a ward for cohort care, resulted in lower mean hospitalisation costs per influenza‐positive and influenza‐negative patient in 2017‐2018 compared to 2016‐2017. Because of low numbers, differences were not significant. The new clinical pathway appeared to improve patient flow and influenza awareness in JBH, which is most clearly illustrated by a relative decrease in admissions and a shorter LOS of patients suspected of influenza.^5^ It has to be noted that differences in ICU admissions and LOS in ICU in 2016‐2017 contributed substantially to the decrease in mean costs of especially influenza‐negative patients. This is primarily caused by the higher number of ICU admissions and longer length of ICU stay in influenza‐negative patients in 2016‐2017 than 2017‐2018.

While most influenza POCT studies focused on test performance, to our knowledge only one study investigated POCT‐associated costs. In contrast to our results, this study in an acute paediatric ward found no change in mean hospitalisation costs for patients with proven influenza infection after the introduction of influenza POCT.^10^ This could be explained by different study populations (children versus adults) and wards (acute ward versus all ward types). Our study has several limitations to take into account. First, our hospitalisation costs are likely to be an underestimation of the true costs, because they are restricted to influenza‐related costs. Additional costs because of treatment of complications were not taken into account. Second, because of data limitations, duration of isolation and type of requested diagnostics were based on literature.

To conclude, this study suggests favourable economic results regarding a new clinical pathway with influenza POCT, reflecting a more efficient care of patients suspected of influenza presenting at the ED. Acknowledging the research gap in influenza POCT‐associated costs, further research into the cost‐effectiveness of influenza POCT is recommended.

## 5 PUBLICATION ETHICS

The Dutch Medical Research Involving Human Subjects Act did not apply to this study, because anonymous data were used and there were no interventions other than routine clinical care. A waiver for full medical ethical review was obtained from the Medical Ethical Committee of the University Medical Center Utrecht (reference number WAG/mb/16/019885) and JBH (reference number 2016.07.06.01).

## CONFLICT OF INTEREST

None declared.

## AUTHOR CONTRIBUTION

**Sierk D. Marbus:** Conceptualization (equal); Formal analysis (equal); Methodology (equal); Writing‐original draft (lead); Writing‐review & editing (lead). **Suzanne PM Lutgens:** Conceptualization (equal); Formal analysis (supporting); Methodology (supporting); Writing‐review & editing (supporting). **Arianne B van Gageldonk‐Lafeber:** Conceptualization (equal); Formal analysis (supporting); Methodology (supporting); Writing‐review & editing (supporting). **Eric HLCM Hazenberg:** Conceptualization (equal); Data curation (lead); Formal analysis (supporting); Methodology (supporting); Resources (lead); Writing‐review & editing (supporting). **Mirjam HA Hermans:** Conceptualization (equal); Formal analysis (supporting); Methodology (supporting); Writing‐review & editing (supporting). **Anita Suijkerbuijk:** Conceptualization (equal); Formal analysis (equal); Methodology (equal); Supervision (lead); Writing‐review & editing (supporting).

## Supporting information

Table S1‐S2

## Data Availability

The data sets generated during and/or analysed during the current study are not publicly available yet, due to legal concerns and ongoing additional research. Data can be made available for peer review on reasonable request through contacting the corresponding author.
