# Supplementary material for: Costs of a clinical pathway with point‐of‐care testing during influenza epidemic in a Dutch hospital
Source: Influenza Other Respir Viruses. 2020 Oct 12;15(2):202–5. doi: 10.1111/irv.12808 (PMC7902253; doi:10.1111/irv.12808)
Supplement: Supplementary file 1 — Table S1‐S2 [file IRV-15-202-s001.docx]

**Supplementary file**

**Table 1** Cost unit prices per cost type expressed in 2018 euros

| **Cost type** | **Cost details** | | **Cost unit price** | **Source** |
| --- | --- | --- | --- | --- |
|  |  |  | € |  |
| Medication  (per treatment) | antibiotics | amoxicillin  cefuroxime  ciprofloxacin  average 2016-2017  average 2017-2018 | 25.60  50.40  0.58  29.40  28.90 | (9) |
|  | oseltamivir | | 21.80 | (9) |
| Emergency department | consultation | | 269.65 | (11) |
| (per admission) | diagnostics  influenza POCT (Cobas Liat)  influenza LDT (BD MAX System)  chest X-ray  blood & urine screening test  urine antigen test | | 112.44  112.44  42.53  51.80  25.72 | (†)  (†)  (13)  (14) |
| Hospitalization  (per day) | general ward  weighted arithmetic mean | | 495.57 | (11) |
|  | intensive care unit | | 2097.85 | (11) |
|  | additional isolation costs | | 15.53 | (15) |

^†^personal communication Jeroen Bosch Hospital

**Table 2** The impact of intensive care unit admissions on hospitalization costs of influenza-positive and influenza-negative patients during influenza epidemic 2016-2017 and 2017-2018

| **Cost type** | **Influenza**  **test result** | **Influenza epidemic 2016-2017**  **(week 48, 2016 – week 10, 2017)** | | | **Influenza epidemic 2017-2018**  **(week 2, 2018 – week 15, 2018)** | | |
| --- | --- | --- | --- | --- | --- | --- | --- |
|  |  | **No. patients** | **Hospitalisation costs**  € | | **No. patients** | **Hospitalisation costs**  € | |
|  |  |  | Per week | Per patient |  | Per week | Per patient |
| **Hospitalization costs, including ICU admissions** | | | | | | | |
| Total costs | positive  negative | 189  402 | 46,125  137,075 | 3,661  5,115 | 624  922 | 86,488  149,141 | 2,4955  2,912 |
| **Hospitalization costs, excluding ICU admissions** | | | | | | | |
| Total costs | positive  negative | 178  375 | 38,432  87,481 | 3,239  3,499 | 603  902 | 74,251  133,214 | 2,216  2,658 |
